# Population control methods in stochastic extinction and outbreak scenarios

**DOI:** 10.1371/journal.pone.0170837

**Published:** 2017-02-02

**Authors:** Juan Segura, Frank M. Hilker, Daniel Franco

**Affiliations:** 1 Departamento de Matemática Aplicada, E.T.S.I. Industriales, Universidad Nacional de Educación a Distancia (UNED), Madrid, Spain; 2 Institute of Environmental Systems Research, School of Mathematics / Computer Science, Osnabrück University, Osnabrück, Germany; São Paulo State University, BRAZIL

## Abstract

Adaptive limiter control (ALC) and adaptive threshold harvesting (ATH) are two related control methods that have been shown to stabilize fluctuating populations. Large variations in population abundance can threaten the constancy and the persistence stability of ecological populations, which may impede the success and efficiency of managing natural resources. Here, we consider population models that include biological mechanisms characteristic for causing extinctions on the one hand and pest outbreaks on the other hand. These models include Allee effects and the impact of natural enemies (as is typical of forest defoliating insects). We study the impacts of noise and different levels of biological parameters in three extinction and two outbreak scenarios. Our results show that ALC and ATH have an effect on extinction and outbreak risks only for sufficiently large control intensities. Moreover, there is a clear disparity between the two control methods: in the extinction scenarios, ALC can be effective and ATH can be counterproductive, whereas in the outbreak scenarios the situation is reversed, with ATH being effective and ALC being potentially counterproductive.

## 1 Introduction

Many populations fluctuate in abundance [1, 2], which may have consequences for their persistence, resilience to perturbations, the attainable yield from exploiting such populations, and which may have knock-on effects on other species and the stability of the ecosystem as a whole [3–5]. Adaptive limiter control (ALC) and adaptive threshold harvesting (ATH) are two control strategies that aim to reduce oscillations in population size. They are related in the sense that their interventions (restocking and harvesting, respectively) only take place if the population size has dropped below or climbed above a certain proportion of its value in the preceding time step, i.e., the threshold population sizes to trigger interventions are ‘adaptive’. ALC has recently been shown to promote population stability experimentally in populations and metapopulations of the fruit fly *Drosophila melanogaster* [6], in numerical simulations as well as by analytical results [6–10]. ATH has been proposed and analyzed in [11].

Both methods have similar stabilizing properties [11], yet they can be expected to be implemented in different biological contexts. As a restocking strategy, ALC is likely to be applied in biological conservation, species re-introduction programmes and the release of biocontrol agents, while ATH as a harvesting strategy is expected to be implemented in pest containment programmes or in the management of species with commercial value (e.g. fisheries). However, on the one hand harvesting strategies can have the counter-intuitive effect of increasing population size, see the review [12]. On the other hand, adding individuals could promote extinction. For example, such an intervention can shift a bistable system to another attractor with larger extinction risk, see e.g. [13], which may be especially the case when the alternative attractor oscillates and the trough values come close to an extinction threshold [14–16]. It is therefore not straightforward to assume that harvesting reduces outbreaks risk nor that restocking reduces extinction risk.

Moreover, all of the models of ALC and ATH have made use of unimodal production curves such as the Ricker map. In reality, control is likely to be necessary when populations are subject to biological mechanisms that put them at risk or promote recurrent population outbreaks. These situations are characterized by bistability such that the population can jump stochastically between two attractors, one of which is less desirable than the other from a control point of view (for nuisance species we want to avoid the high-density attractor and for endangered species we want to avoid the small-density attractor or extinction state). Biological mechanisms inducing bistability have been largely ignored, however, with the exception of ALC models setting small populations to zero with a fixed probability [6, 9] and ATH models considering strong Allee effects in some numerical experiments [11].

In this paper, we study ALC and ATH systematically in two different population contexts. In the first one, populations are vulnerable to extinction due to a strong Allee effect. The Allee effect is a positive density dependence at low population sizes that occurs when the individual fitness increases with the number of individuals [17]. If the Allee effect is strong, there is bistability and small populations go extinct due to a lack of conspecifics (which may be caused by difficulties in finding mates or in cooperation, for instance). We will consider three different extinction scenarios. In the first one, populations monotonically decline before going extinct. In the second one, populations grow to a large population size and then collapse due to overcompensation. In the third one, a strong Allee effect interacts with population cycles and causes essential extinction [14–16]. This happens when the fluctuating population drops below the minimum viable population size set by the Allee effect. The transition to essential extinction occurs through a boundary collision, and thus environmental changes may cause abrupt population collapses. As ALC and ATH reduce the fluctuation range, their stabilizing properties are particularly interesting in this extinction scenario.

Allee effects have been empirically found in many species including mammals and birds [18], plants [19], insects [20] and marine invertebrates [21], and their relevance is particularly recognized in conservation biology [17, 22–24]. However, Allee effects have also been detected in a large number of invasive species like the gypsy moth *Lymantria dispar* [25, 26], the zebra mussel *Dreissena polymorpha* [27] or the pine sawyer *Monochamus alternatus* [28]. Thus, Allee effects are relevant not only for the survival of endangered populations but also in the prevention of outbreaks.

Outbreaks are the second population context considered in this paper. Here, we study two different outbreak scenarios. The first one is based on a strong Allee effect model, with extinction being the non-outbreak state. In the second scenario the non-outbreak state is positive. It is based on a gypsy moth outbreak model that combines density-dependent regulation by predation with host–pathogen dynamics [29]. This causes multistability between a high-density and a low-density attractor, the latter of which may be more complex and even chaotic. With stochastic perturbations ubiquitous in nature, the model population jumps rather unpredictably between different states. Again, as ALC and ATH tend to reduce fluctuation ranges, they might abate transitions to outbreak states. The importance of stochasticity is also well recognized in biological invasions [25, 30] and for endangered species [24, 31–33].

In the next Section, we introduce the mathematical models that describe first the underlying population dynamics in the absence of any control and then the two control methods of ALC and ATH. Section 3 analyzes the effect of the control methods on deterministic and stochastic populations in three different extinction scenarios. We then turn our attention to the two outbreak scenarios in Sections 4 and 5. Section 6 draws conclusion on the applicability of ALC and ATH in the biological contexts considered.

## 2 Population dynamics and control methods

We start by describing the underlying population dynamics in the absence of control, to be followed by a description of the two control methods.

### 2.1 Deterministic and stochastic population models

We assume that the population dynamics are described by a first-order difference equation of the form

$$\begin{array}{c} x_{t+1}=f\left(x_{t}\right),\;x_{0}\in \left[0,+\infty \right),\;t\in N, \end{array}$$

where *x*_*t*_ denotes the population size at generation *t* and *f*: [0, +∞) → [0, +∞) is the population production function or the stock–recruitment curve. We assume that the population has a strong Allee effect and that there are three fixed points, namely the extinction state *x* = 0, the Allee threshold *L* > 0 and an equilibrium *K* > 0 corresponding to the carrying capacity. Moreover, the population dynamics are assumed to be overcompensatory such that the stock–recruitment curve is unimodal with a long tail, peaking at *x* = *d*.

These biological assumptions can be expressed mathematically in the following conditions on the map *f*:

(C1) *f* is continuously differentiable and such that *f*(0) = 0, *f*′(0^+^) > 0 and *f*(*x*) > 0 for all *x* ∈ (0, +∞).
(C2) *f* has three non-negative fixed points *x* = 0, *x* = *L* > 0 and *x* = *K* > *L*, with *f*(*x*) > *x* for *x* ∈ (*L*, *K*) and *f*(*x*) < *x* for *x* ∈ (0, *L*) ∪ (*K*, +∞).
(C3) *f* has a unique critical point *d* ∈ (0, *K*) such that *f*′(*x*) > 0 for all *x* ∈ (0, *d*) and *f*′(*x*) < 0 for all *x* ∈ (*d*, +∞).

For numerical simulations, we will consider a population map satisfying (C1)–(C3) that was studied in [14] as a model of mate limitation [22, 34, 35]. On the basis of the Ricker model, a strong Allee effect is induced by the introduction of density dependence in the form

$$\begin{array}{c} f\left(x\right)=x\cdot \exp\left(r\left(1-x/\tilde{K}\right)\right)\cdot I\left(x\right), \end{array}$$ (1)

where *I*(*x*) = *sx*/(1 + *sx*) is the probability of finding a mate, *s* > 0 measures an individual’s searching efficiency and *r*, $\tilde{K}>0$ represent the growth parameter and the carrying capacity for the Ricker model in the absence of mate limitation, respectively. This model and its dynamics are described in more detail in [14] and [33]. For given values of *r* and $\tilde{K}$, condition (C2) is satisfied only for values of *s* above a certain threshold, below which the population goes asymptotically extinct for all initial conditions.

Deterministic population models like Eq (1) ignore, in some sense, the unpredictability of nature. In order to take into account the effect of random events on the population dynamics, we will introduce stochasticity in the underlying model. Since Allee effects are expected to operate on small populations, we focus our attention on demographic stochasticity. One way to include this is

$$\begin{array}{c} x_{t+1}=f\left(x_{t}\right)\cdot \exp\left(\sqrt{\frac{\sigma^{2}}{f(x_{t})}}\cdot \varepsilon_{t}-\frac{\sigma^{2}}{2f(x_{t})}\right), \end{array}$$ (2)

which was proposed by [36]. Here, *f* denotes the production function of the deterministic model, *ε*_*t*_ is a normally distributed variable with expectation 0 and variance 1, and parameter *σ* measures the intensity of noise.

### 2.2 Modelling control by adaptive limiters

When a population is controlled by ALC, the intervention is triggered whenever the number of individuals after reproduction, *f*(*x*_*t*_), drops below a certain fraction of its value in the current generation, and individuals are restocked by such an amount that the population size is restored back to that fraction. The dynamics of populations controlled by ALC can be described by the difference equation

$$\begin{array}{c} x_{t+1}=\max\left\{f\left(x_{t}\right),c\cdot x_{t}\right\}, \end{array}$$

where *f* is the production function of the uncontrolled population, and *c* ∈ (0, 1) measures the restocking intensity, for more details see [7].

Control by ATH is activated when the number of individuals after reproduction, *f*(*x*_*t*_), has grown and exceeds a certain proportion (>1) of its value in the current generation. Control can take the form of harvesting and sets back the population size to *x*_*t*_/*h*. We assume *h* ∈ (0, 1) such that the proportion is greater than the current population size and harvesting takes place whenever the population has grown below that proportion. Biologically, parameter *h* measures the harvesting intensity, for more details see [11]. Populations controlled by this method can be described by the difference equation

$$\begin{array}{c} x_{t+1}=\min\left\{f\left(x_{t}\right),x_{t}/h\right\}. \end{array}$$

## 3 Preventing extinction

We will study three different extinction scenarios related to the Allee effect. In the first one, populations become too small, and in the second one populations become too large (as they collapse below the Allee threshold due to overcompensation). In the third scenario, populations become too cyclic (in the sense that a boundary collision causes essential extinction).

The first two scenarios are related to bistable dynamics induced by the strong Allee effect. The existence of bistability in a deterministic population means that there is a minimum viable number of individuals *L* (the Allee threshold) below which the population goes extinct and above which it persists for a set of initial conditions with positive Lebesgue measure. All the results stated in this Section for deterministic populations (both controlled and uncontrolled) are proven in the Appendix.

### 3.1 Small population extinction

Small deterministic populations with a size below *L* eventually go extinct. This is the reason why populations with a strong Allee effect are considered particularly vulnerable to extinction. This vulnerability can be expressed in terms of different statistics, such as the extinction probability, the first passage probability or the mean time to extinction [24]. We will use the first of these measures to study the effect of ALC and ATH on population persistence.

**Fig 1:**
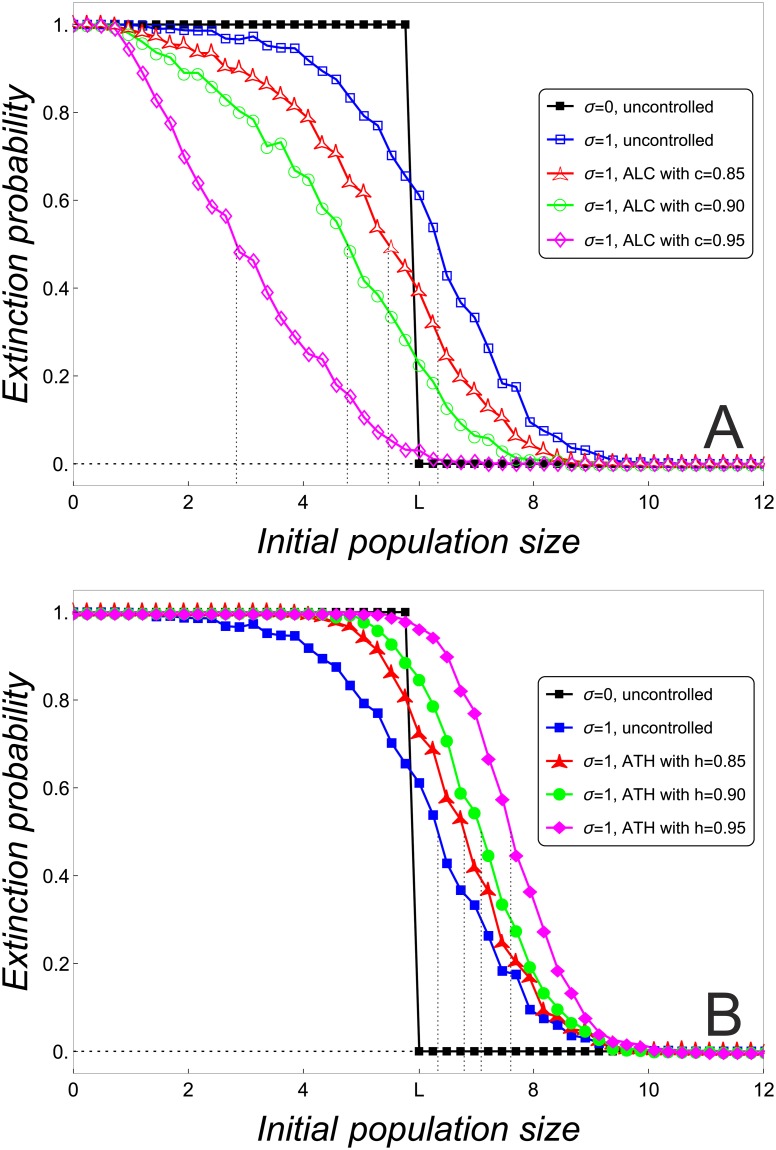
Probability of extinction of small stochastic populations. Probability of extinction in terms of the initial population size around *L* for deterministic (*σ* = 0) and demographic stochastic (*σ* = 1) uncontrolled populations and populations controlled with different intensities by ALC (A) and ATH (B). Calculations are based on Models (1) and (2) with *r* = 4.5, $\tilde{K}=400$ and *s* = 0.002 (*L* ≈ 6.015). For a given initial population size, the probability of extinction has been obtained for the first 100 generations and over 1000 replicates.

Before doing so, we consider uncontrolled populations. In the deterministic case, the probability of extinction as a function of the initial population size has the shape of a staircase near the Allee threshold *L*: it equals 1 on the left-hand side of *L* and 0 on the right-hand side of *L* (Fig 1). When stochasticity is taken into account, populations of a small size can be ‘saved’ from impending extinction by random events that occasionally increase the number of individuals above *L*. Conversely, populations that would persist in a deterministic world can fall below *L* due to the effect of noise and eventually go extinct [24]. As a result, stochasticity reduces the abruptness of the deterministic Allee threshold [22, 24, 31], and the extinction probability for stochastic systems has a sigmoid decreasing shape, as shown in Fig 1. This has an important consequence. The concept of an Allee threshold, which for deterministic systems corresponds to the smallest positive fixed point, must be redefined in the case of stochastic populations. This will be done next.

#### 3.1.1 Stochastic Allee threshold

There are two approaches to define the Allee threshold in stochastic models, which we will refer to as stochastic Allee threshold. The first one defines the Allee thresold as the population size corresponding to the inflection point of the sigmoidally decreasing population extinction probability [22, 31]. The second approach defines the Allee threshold as the population size for which the probability of extinction and the probability of persistence are equal [24, 37]. This is also the definition we will use in this paper, as it is practically easier to calculate.

It should be noted though that the two approaches yield different values for the Allee threshold, which can also be seen in Fig 1. Nevertheless, when we know there is always a strong Allee effect present, both approaches result in the same trends as the corresponding values are positively correlated. However, if there is weak or no demographic Allee effect, there is obviously no Allee threshold in the deterministic model and using the second approach could be misleading.

Before investigating the impact of control on the stochastic Allee thresholds, we consider the uncontrolled case. The example in Fig 1 shows that the stochastic Allee threshold (blue curve) is larger than the deterministic Allee threshold *L*. This matches the general consensus that noise renders populations with strong Allee effect more vulnerable to extinction [17, 23, 24, 31, 33].

#### 3.1.2 Controlling small deterministic populations

Let us now analyze the effect of ALC and ATH on small deterministic populations. There are two questions that immediately come to mind. Firstly, can control be beneficial in the sense of saving populations that are doomed to extinction? Secondly, can control be counterproductive in the sense of inducing essential extinction of those populations that might survive otherwise? Proposition 1 in the Appendix shows that neither of these situations ever happen. In particular, neither ALC nor ATH change the extinction probability of deterministic populations around *L*.

**Fig 2:**
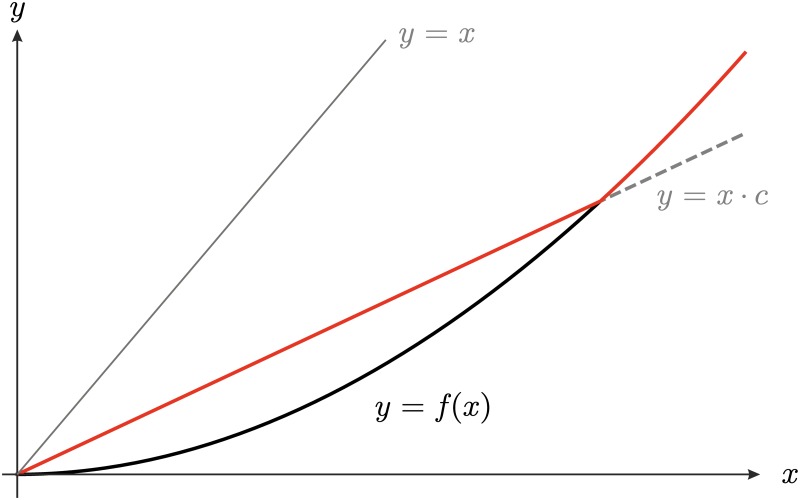
ALC can slow down the convergence to extinction of small deterministic populations. The black curve corresponds to the production function of the uncontrolled Model (1) with *r* = 4.5, $\tilde{K}=400$ and *s* = 0.002, and the red curve to the population controlled by ALC with intensity *c* = 0.5.

Yet, there are slight differences between the effect of the two control methods in such populations. On the one hand, ATH is completely ineffective for small populations because it does not alter the production function around *L*. On the other hand, ALC does change the dynamics around the extinction state from *x*_*t*+1_ = *f*(*x*_*t*_) to *x*_*t*+1_ = *x*_*t*_ ⋅ *c* > *f*(*x*_*t*_) for intensities *c* > *f*′(0^+^). Thus, ALC is able to slow down the extinction process, see Fig 2.

#### 3.1.3 Controlling small stochastic populations

When stochasticity is taken into account, important differences between the effect of ALC and ATH on small populations emerge. ALC with high intensities promotes population persistence by reducing both the probability of extinction and the stochastic Allee threshold (Fig 1A). Basically, there are two reasons for this effect. Firstly, restocking due to ALC can partially mitigate population declines that are caused by noise and that could spur extinction. Secondly, ALC prolongs for *c* > *f*′(0^+^) the transients to the extinction state of deterministic populations that start or drop below *L* (cf. Fig 2). These longer transients increase the chance of stochastic populations to be positively affected by noise and thus be saved for some time.

Under ATH, both the probability of extinction and the stochastic Allee threshold increase with higher harvesting intensities (Fig 1B). Therefore, unlike ALC, ATH seems to be counterproductive to protecting small population. Again, two reasons help to explain this effect. Firstly, ATH is not able to slow down fortuitous declines in the size of populations that start or drop below the Allee threshold. Secondly, the harvesting of ATH tends to reduce any random growth that could move the population away from the extinction state.

#### 3.1.4 Impact of stochasticity and Allee effects

We now investigate how the effect of control on population persistence depends on the level of noise, *σ* on the one hand and the strength of the Allee effect, *s* on the other hand. To this end, we seek to represent the relationship between extinction probability and initial population size in a single quantity. Fig 1 suggests that, for given values of *s* and *σ*, the stochastic Allee threshold is positively correlated to the probability of extinction in terms of the control intensity: those control intensities with a higher extinction probability have a larger stochastic Allee threshold. Hence, we will capture the effect of the control methods on the extinction probability by analyzing the stochastic Allee threshold.

**Fig 3:**
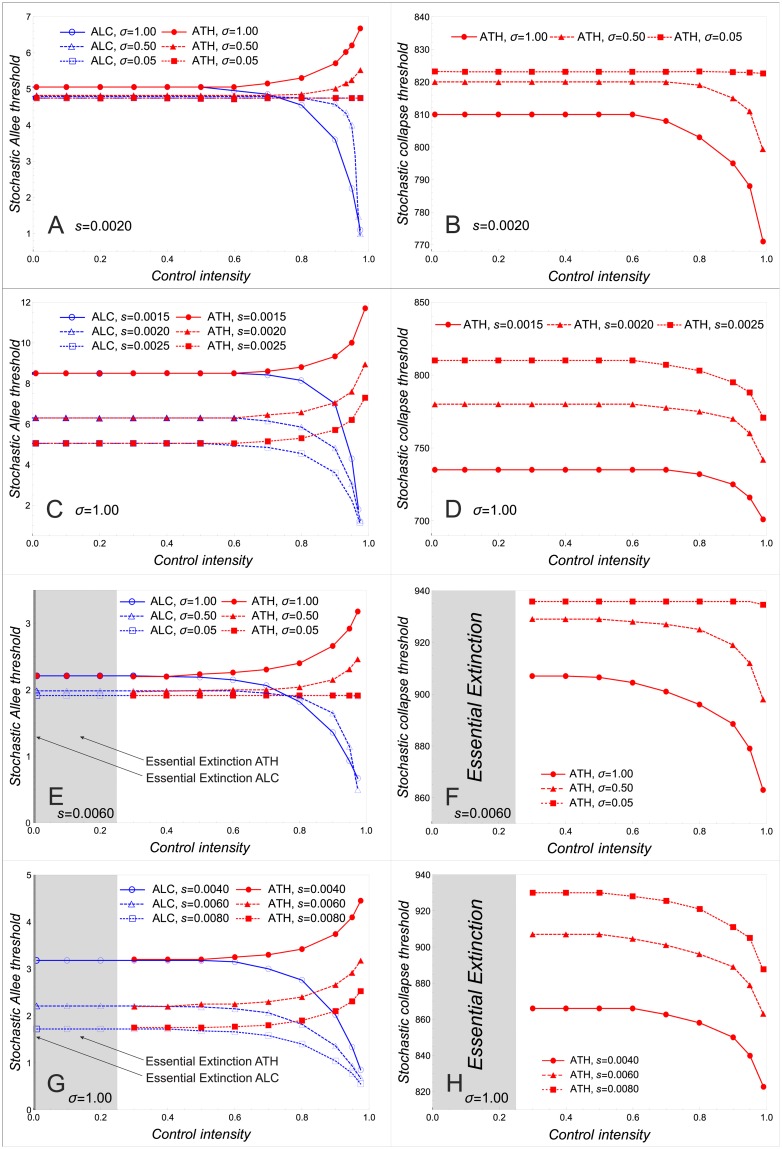
Stochastic Allee and collapse thresholds. Stochastic Allee and collapse thresholds as functions of control intensity for different levels of noise and for different strengths of the Allee effect in the range of bistable dynamics (A to D) and in the range of essential extinction (E to H). Calculations are based on Models (1) and (2) with *r* = 4.5 and $\tilde{K}=400$. For a given initial population size, the probability of extinction has been obtained for the first 100 generations and over 5000 replicates. The right-hand side panels show stochastic collapse threshold only for ATH since they exist under ALC only for extremely small intensities.

Fig 3A shows how the stochastic Allee threshold varies with different levels of noise in the range of bistable dynamics. When the level of noise is low, control exerted by ALC or ATH does not alter the extinction probability and, in this respect, controlled populations behave as the uncontrolled ones. For medium and high levels of noise, differences between controlled and uncontrolled populations arise: on the one hand, for small control intensities, neither ALC nor ATH alter the extinction probability; on the other hand, for medium and large control intensities, extinction probability is reduced by ALC and increased by ATH. This disparity between control methods (i) becomes more pronounced and (ii) starts to arise at smaller control intensities as the noise level increases. These observations corroborate and extend the results in Fig 1.

Different strengths of the Allee effect influence, of course, the quantitative level of the stochastic Allee threshold (Fig 3C). However, they affect neither the magnitude of disparity between ALC and ATH nor the minimum control intensity for which disparity between ALC and ATH appears.

#### 3.1.5 Summary

In deterministic systems, neither ALC nor ATH are effective in changing the vulnerability of small populations to extinction associated to a strong Allee effect. The same holds true in stochastic systems with low levels of noise. If the population is sufficiently noisy, the control effect depends on the control intensity. For small control intensities, ALC and ATH are still ineffective. For medium and large control intensities, however, there is a clear difference between the control methods. While ALC decreases the stochastic Allee threshold and thus promotes population persistence, ATH decreases the stochastic Allee threshold and thus increases the risk of extinction.

### 3.2 Large population extinction

**Fig 4:**
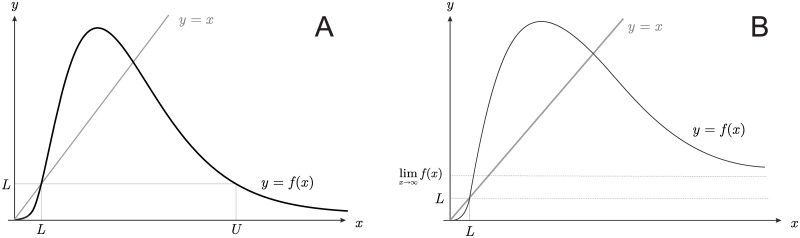
Large population extinction. Large populations can be driven to extinction if there is a collapse threshold *U* and population size exceeds that threshold. A: The collapse threshold exists if lim_*x*→+∞_*f*(*x*) < *L*. Then there is a *U* such that *f*(*x*) > *L* for all *x* ∈ (*L*, *U*) and *f*(*x*) < *L* for all *x* ∈ (*U*, +∞). B: There is no collapse threshold if lim_*x*→+∞_*f*(*x*) ≥ *L*, because then *f*(*x*) > *L* for all *x* > *L*.

When a population is subject to a strong Allee effect, conservation concerns usually seem to focus on small populations. However, in the presence of overcompensation, also large populations can be vulnerable to extinction. Under assumptions (C1)–(C3), this happens when the limit of *f* for *x* → ∞ is below *L* (this is the case for the mate-finding Allee effect Model (1) considered here, for which that limit is 0). Under these conditions, there exists a *collapse threshold* *U* > *K* such that deterministic uncontrolled populations with a number of individuals above it go eventually extinct (see Fig 4A). By contrast, if the limit of *f* for *x* → ∞ is greater than *L*, all populations starting in (*L*, +∞) persist (see Fig 4B).

#### 3.2.1 Controlling large deterministic populations

Assuming that a collapse threshold *U* exists, we now analyze how the control methods affect the extinction risk of large deterministic populations. We start by noting that ATH does not alter the production function around *U*. Hence, this method has no effect on the vulnerability of large deterministic populations.

**Fig 5:**
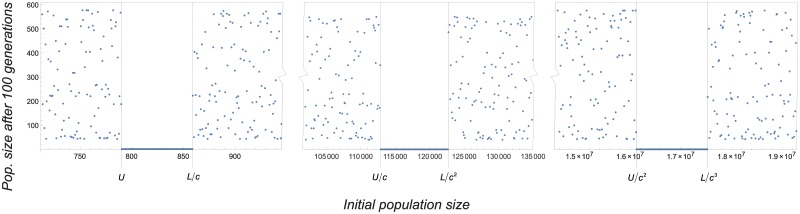
Persistence and extinction depend on the initial population size for ALC. Deterministic population sizes over the first 100 generations as a function of the initial value for ALC. Parameter values *K* = 400, *r* = 4.5, *s* = 0.002, *σ* = 0 (deterministic), and *c* = 0.007 (the value of *L*/*U* is 0.007614).

By contrast, ALC can suppress that vulnerability. Proposition 1 shows that this happens only partially for control intensities *c* < *L*/*U*, since populations with sizes in

$$\begin{array}{c} \left[L/c,U/c\right]\cup \left[L/c^{2},U/c^{2}\right]\cup \left[L/c^{3},U/c^{3}\right]\cup \cdots \end{array}$$

persist, while populations with sizes in

$$\begin{array}{c} \left(U,L/c\right)\cup \left(U/c,L/c^{2}\right)\cup \left(U/c^{2},L/c^{3}\right)\cup \cdots \end{array}$$

asymptotically go extinct (cf. Fig 5). For intensities *c* > *L*/*U*, ALC excludes extinction of large populations as all populations with sizes in [*L*, +∞) persist.

Regarding the critical control intensity *L*/*U*, it is remarkable that its value is less than 0.15 for the mate-finding Allee effect model considered here (for all values of *s* for which the system exhibits bistable dynamics and for all values of *r* in the interval (1, 6)). In view of this and if the restocking intensity is greater than this value in practical implementations, one may assume that ALC totally cancels the effect of overcompensation on population persistence for the deterministic model.

#### 3.2.2 Controlling large stochastic populations

Similarly to the stochastic Allee threshold, we need to extend the concept of the collapse threshold to systems that include noise. To this end, we note that, in deterministic uncontrolled systems, the extinction probability for large population sizes around *U* is switch-like: it equals 0 on the left-hand side of *U* and 1 on the right-hand side (not shown here). Noise can shift the number of individuals from one side of *U* to the other, thus conferring a sigmoid shape to the extinction probability around that point (not shown here). This allows us to define the *stochastic collapse threshold* as the population size for which the extinction probability equals the persistence probability.

Let us first consider the effect of ALC. Populations controlled by this method have a zero extinction probability around *U* (not shown here). Consequently, there is no stochastic collapse threshold for these populations. This is consistent with the fact that ALC with large enough intensity diminishes extinction risk in the deterministic model.

By contrast, populations controlled by ATH have an extinction probability of sigmoid shape around *U*. Thus, we can study the effect of ATH by analyzing the stochastic collapse threshold (Fig 3B and 3D). For low levels of noise, increasing ATH intensity does not change the stochastic collapse threshold (Fig 3B and 3D). For medium and high levels of noise, we observe the following: (i) The stochastic collapse threshold and thus population persistence become smaller with higher noise levels. (ii) There is a critical control intensity, beyond which increasing ATH intensity drastically deteriorates population persistence. (iii) This critical control intensity becomes smaller, the higher the level of noise.

Fig 3D shows that the stochastic collapse threshold decreases with the strength of the Allee effect. This makes sense as a collapse is more likely the stronger is the Allee effect. For low control intensities, ATH does not change the stochastic collapse threshold. For medium and high control intensities, ATH promotes population collapses. The stronger the Allee effect, the sooner the onset of the deteriorating effect of ATH.

#### 3.2.3 Summary

Regarding the collapse of large populations, there is a clear difference between the control methods. ALC with high enough a restocking intensity ensures the survival of deterministic populations with a large number of individuals that would be doomed to extinction in the absence of control. In stochastic systems, ALC completely prevent collapses of large populations considered here. By contrast, ATH is either ineffective (in deterministic systems and for small control intensities in stochastic systems) or counterproductive (for medium and high control intensities in stochastic systems).

### 3.3 Essential extinction

In the previous two extinction scenarios, the deterministic uncontrolled population dynamics are bistable, i.e. the fate of the population depends on the initial condition. Now we consider the scenario of essential extinction, where the only attractor is the extinction state. This means that populations go extinct with probability 1 for randomly chosen initial conditions.

#### 3.3.1 Deterministic population dynamics

It is in the scenario of essential extinction that we find the main advantage of ALC and ATH: both methods can induce bistability and thus facilitate population persistence if the control intensity is greater than a critical threshold. This is proven in Proposition 2 in the Appendix. The critical thresholds for the control intensities are *c*_0_ = *L*/*U* for ALC and *h*_0_ = *d*/*f*(*d*) for ATH. Once the critical control intensity has been exceeded and the controlled system exhibits bistability, populations behave as described in the previous two scenarios.

#### 3.3.2 Stochastic population dynamics

While noise in bistable systems can be occasionally beneficial to populations by perturbing their size above the extinction threshold, this can never happen in the scenario of essential extinction, see also Corollary 4.3 in [33]. Deterministic populations showing essential extinction only persist for a small number of initial conditions, in particular for those that coincide with the positive fixed points. Yet, when noise is taken into account, stochastic uncontrolled populations go extinct for all possible initial sizes, including the positive fixed points, as random events perturb the population size from equilibrium. Hence, noise is in this respect counterproductive.

Let us now study the effect of ALC and ATH on stochastic systems for which the deterministic dynamics exhibits essential extinction. As in the previous two extinction scenarios, we will analyze the stochastic Allee thresholds and the stochastic collapse thresholds (Fig 3E to 3H).

As in the deterministic setting, both ALC and ATH are able to save stochastic populations that would be doomed to essential extinction in the absence of control. This can be seen in Fig 3E to 3H by the existence of a stochastic Allee or collapse threshold. The control-mediated survival occurs if the control intensity exceeds a critical value; in the case of ALC, the critical control intensity is close to (but not exactly) zero; i.e. without control there would be essential extinction. Once the control intensity of ALC or ATH exceeds the corresponding critical value, the population becomes bistable and the effect of the control methods on stochastic populations is analogous to the case of bistable dynamics. The only remarkable difference is that the disparity between populations controlled by ALC on the one hand and ATH on the other hand arises for somewhat lower control intensities than in the bistable scenarios (cf. Fig 3A and 3C with Fig 3E and 3G).

## 4 Preventing population outbreaks

The previous Section was concerned with population extinction. Now we shift attention from vulnerable species to pests, and the aim is to contain their population size in order to avoid outbreaks. We will study bistable populations only, because under essential extinction there appears to be less need for controlling outbreaks.

### 4.1 Outbreaks and probability of outbreaks

First, we need to specify what exactly we mean by outbreaks. In the literature, there are different definitions of outbreaks, many of which concern a specific situation or population model, e.g. [29, 38, 39]. In our case, if *K* is stable, the concept of an outbreak does not make sense because all orbits are monotonically attracted towards this point or towards the extinction state. There are no sustained oscillations in population size, which could be controlled by ALC or ATH. Hence, we restrict our attention to *K* being unstable, such that all populations not attracted towards the extinction state oscillate in size around *K*. In this situation, an obvious definition of outbreaks is related to the amplitude of these oscillations. Hence, we will consider as *outbreak* any population size exceeding the midpoint between the unstable fixed point *K* and the maximum population size *f*(*d*).

**Fig 6:**
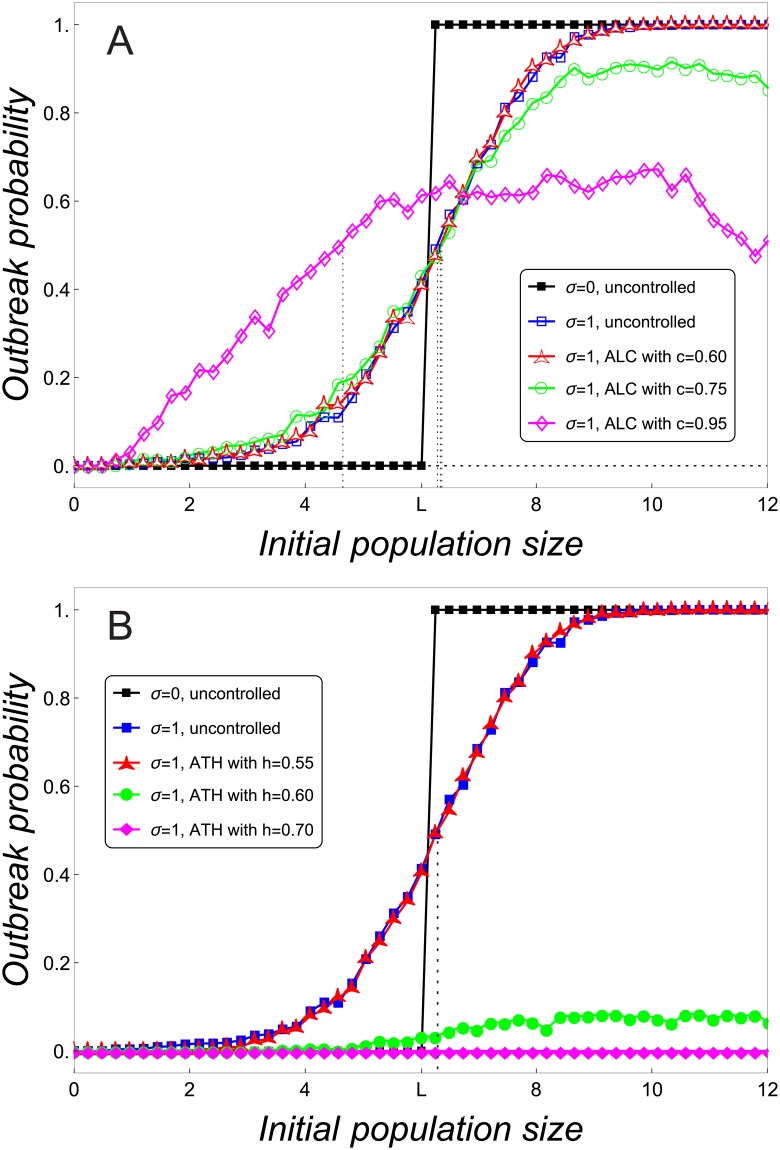
Probability of outbreak in terms of the initial population size. The population is controlled by (A) ALC and (B) ATH. Population dynamics are deterministic (*σ* = 0) or with demographic stochasticity (*σ* = 1). Calculations are based on Models (1) and (2) with *r* = 4.5, $\tilde{K}=400$ and *s* = 0.002 (*L* ≈ 6.015). Population outbreaks are considered to occur when the number of individuals exceeds (*K* + *f*(*d*))/2. For a given initial population size, the outbreak probability has been obtained for the first 100 generations and over 1000 replicates.

For uncontrolled and deterministic populations, Fig 6 shows that the probability of outbreaks switches at the Allee threshold *L*: for initial population sizes below (above) *L* the outbreak probability is zero (one). When stochasticity is included, we observe an effect reverse to that for the extinction probability: noise can cause booms in stochastic uncontrolled populations that start or drop below *L*, while populations starting above *L* can remain below the outbreak threshold thanks to random declines caused by noise. This confers a sigmoid shape to the outbreak probability (but mirrored horizontally in comparison to the extinction probability). The stochastic outbreak threshold, i.e. the population size at which the probability of an outbreak equals the probability of no outbreak, increases in comparison to the deterministic outbreak threshold in Fig 6. Hence, stochasticity seems to render populations with a strong Allee effect more prone to outbreaks.

### 4.2 Effect of ATH

Fig 6B shows that ATH tends to reduce the probability of outbreaks. This happens for sufficiently large control intensities (*h* ≳ 0.55) and can be easily explained by the harvesting action of ATH that can mitigate any fortuitous population growth due to noise. For control intensities that are too small, there is no difference in the outbreak probability between controlled and uncontrolled populations.

Moreover, Fig 6B shows that, for high control intensities (*h* ≳ 0.7), ATH completely prevents population booms. This may be explained as follows. For sufficiently high control intensities, ATH establishes in a deterministic system bistability between zero and a trapping region around *K* (see Proposition 2 in the Appendix). As a consequence, the trapping region imposes an upper bound on the population size. This upper bound decreases with control intensity and tends to *K* when *h* → 1. Consequently, for high enough control intensities the number of individuals is asymptotically bounded by a value that is below the outbreak threshold. Hence, population booms are unlikely to happen. They can only occur if the effect of noise moves the population size above the trapping region for the deterministic system in such a way that the outbreak threshold is exceeded.

### 4.3 Effect of ALC

Comparing Fig 6A with Fig 6B reveals that ALC has different impacts on outbreak probabilities than ATH has. First we note that for small and medium control intensities (*c* ≲ 0.6), ALC seems to change outbreak probabilities only marginally. This behaviour may be explained by two opposing effects of ALC. On the one hand, the restocking of ALC mitigates any random population decline, which tends to promote the risk of outbreaks. On the other hand, also ALC establishes a trapping region around *K* for large enough control intensities (Proposition 2 in the Appendix). Then there is an asymptotic upper bound on population size, and this bound decreases with control intensity. For small and medium control intensities, the upper bound is large and potentially greater than the outbreak threshold, while the capability of ALC to restock population declines is weak.

For high control intensities, ALC has a very different effect than ATH. We have to distinguish between small and large initial population size. For initial population sizes above *L*, ALC significantly reduces the outbreak probability. This is probably due to the asymptotic upper bound. However, ALC cannot completely prevent outbreaks (cf. the magenta curve for *c* = 0.95) as ATH can, which is probably due to the restocking. For initial population sizes below *L*, ALC can have a counterproductive effect, as ALC increases extinction probability in comparison to the uncontrolled population. This happens approximately for *c* ≥ 0.6 (cf. green and red curves with the uncontrolled curve in Fig 6A). For very large control intensities, ALC increases extinction risk of population sizes below *L* drastically (e.g., for *c* = 0.95 in Fig 6A). This may be caused by the capability of ALC to offset population declines, which becomes so strong for high control intensities such that populations are almost fully shielded against random declines. However, they benefit from all possible random growths, which inflates outbreak risk.

### 4.4 Summary

ATH reveals itself as especially suitable for the control of nuisance species as it reduces or completely prevents stochastic outbreaks of small populations. By contrast, ALC tends to be ineffective for low and medium control intensities and is counterproductive for high control intensities.

## 5 Controlling outbreaks of forest-defoliating insects

In the previous Section we have considered outbreaks in a bistable population with a strong Allee effect. In this particular setting, one of the two attractors is the extinction state. That is, if the population has gone extinct, there cannot be any outbreak in the following generation unless there is immigration, invasion or some form of external perturbation. However, in bistable situations where both attractors are positive, the population can ‘rest’ in a low-density state until an outbreak is triggered by some mechanism and the population bursts to a higher-density attractor.

Such a situation often occurs in models of forest-defoliating insects. Here, we consider a model by Dwyer *et al*. [29] that incorporates the effect of a generalist predator to a classical host–pathogen system. The non-dimensionalized stochastic version of this model reads

$$\begin{array}{c} 1-I\left(x_{t},z_{t}\right)={\left(1+\frac{1}{k}\left(x_{t}I\left(x_{t},z_{t}\right)+z_{t}\right)\right)}^{-k},\\ x_{t+1}=\lambda x_{t}\left(1-I\left(x_{t},z_{t}\right)\right)\left(1-\frac{2ABx_{t}}{B^{2}+x_{t}^{2}}\right)\varepsilon_{t},\\ z_{t+1}=\phi x_{t}I\left(x_{t},z_{t}\right), \end{array}$$ (3)

where the two variables *x*_*t*_ and *z*_*t*_ represent the host and pathogen densities in generation *t*, respectively. Given these densities, *I*(*x*_*t*_, *z*_*t*_) is the fraction of infected hosts. The term *ε*_*t*_ is a log-normal random variable with median 1 and standard deviation *σ*. Regarding the parameters in this model, λ represents the net defoliator fecundity, *ϕ* is the between-season impact of the pathogen, *A* is the maximum fraction of defoliators killed by the predator, *B* is the ratio of the density at maximum predation to the epidemic threshold and *k* is the inverse squared coefficient of variation of the transmission rates, which follows a gamma distribution. Parameter values have been estimated for populations of the gypsy moth *Lymantria dispar* as the host (defoliator) and a baculovirus as the pathogen, yielding λ = 74.6, *ϕ* = 20, *A* = 0.967, *B* = 0.14, and *k* = 1.06 [29]. For these values, the deterministic model has three equilibria with high, intermediate and low defoliator densities. At the high-density equilibrium the defoliator is controlled by the pathogen while the predator is relatively unimportant. This equilibrium is unstable and induces an oscillatory attractor. The low-density equilibrium is stable and the control over the defoliator is exerted by the predator, with the influence of the pathogen being fairly irrelevant. Finally, the intermediate-density equilibrium is unstable. The inclusion of stochasticity makes the defoliator density move unpredictably among attractors and induces high variability in the time between insect outbreaks.

Since our goal is to diminish outbreaks of the defoliator population, we consider control actions on the state variable *x*_*t*_ only. Then, a model including ALC can be described by modifying the second equation of Eq (3) to

$$\begin{array}{c} x_{t+1}=\max\left\{\lambda x_{t}\left(1-I\left(x_{t},z_{t}\right)\right)\left(1-\frac{2ABx_{t}}{B^{2}+x_{t}^{2}}\right),c\cdot x_{t}\right\}\varepsilon_{t}. \end{array}$$

Similarly, for ATH we obtain

$$\begin{array}{c} x_{t+1}=\min\left\{\lambda x_{t}\left(1-I\left(x_{t},z_{t}\right)\right)\left(1-\frac{2ABx_{t}}{B^{2}+x_{t}^{2}}\right),x_{t}/h\right\}\varepsilon_{t}. \end{array}$$

**Fig 7:**
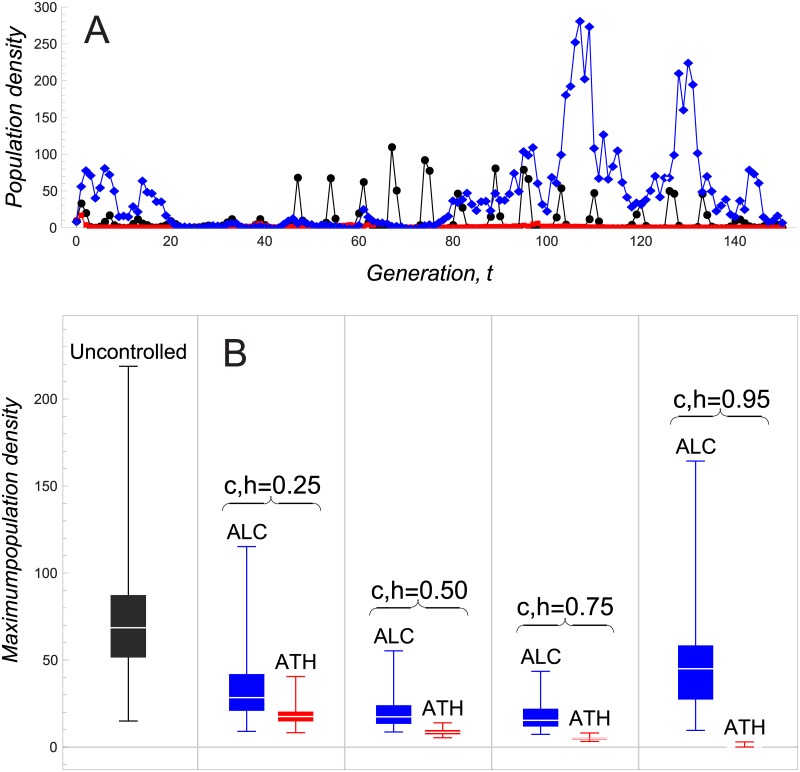
Numerical simulations for the gypsy moth model. (A) Comparison of model time series for populations of the defoliator gypsy moth. The black curve corresponds to the uncontrolled system, the blue one to the system controlled by ALC with *c* = 0.9 and the red one to ATH with *h* = 0.9. (B) Box plots of the maximum population density of defoliators for the uncontrolled system and systems controlled by ATH and ALC with different intensities. Calculations are based on Model (3) with λ = 74.6, *ϕ* = 20, *A* = 0.967, *B* = 0.14, *k* = 1.06 and *σ* = 0.5. Initial densities in (A) are *x*_0_ = 10 for the defoliator and *z*_0_ = 7 for the pathogen. Values in (B) have been obtained from 100 time series with initial population densities uniformly distributed in [0.01, 100] and a time horizon of 50 generations.

Fig 7A shows time series of three stochastic defoliator populations with the same initial conditions corresponding to the uncontrolled system and systems controlled by ALC and ATH with intensities *c* = *h* = 0.9. ATH keeps the defoliator densities close to zero for the entire time period considered. By contrast, under the control of ALC, the defoliator reaches densities much higher than in the absence of control.

In order to analyze if this is always the case, Fig 7B compares the maximum defoliator densities for the uncontrolled and controlled populations with different control intensities and different initial conditions for both pathogen and host. We have chosen maximum densities as they are the quantity of interest in outbreak situations. ATH clearly reduces the maximum defoliator density for the range of control intensities considered. By comparison, maximum population sizes are both higher and more variable when controlled by ALC and ATH. In particular, while ATH performs better in reducing population maxima when increasing control intensities to the values shown in (Fig 7B), ALC loses some of its effectiveness for the control intensity of *c* = 0.95. This can also be seen in Fig 7A, where the maximum density of populations controlled by ALC is much larger than when the number of insects is not controlled.

## 6 Discussion and conclusions

We have compared the impact of ALC and ATH on extinction and outbreak probabilities. In order to capture stochastic effects, we have used the concept of stochastic Allee thresholds, stochastic collapse thresholds and stochastic outbreak thresholds. Both control methods have in common that they become effective (in the sense of changing stochastic extinction or outbreak thresholds) only for sufficiently large control intensities. If their interventions do show an effect, there is a clear disparity between the two methods in each of the biological situations considered.

Regarding the control of outbreaks, we have studied how the control methods affect the outbreak probabilities. ATH proves beneficial in terms of reducing or even completely eliminating outbreak probability. It can also significantly curtail the magnitude of population booms (measured by maximum defoliator population sizes in the gypsy moth model). By contrast, ALC is either ineffective or even counterproductive. This holds for both the Allee effect and the gypsy moth model. As ATH removes individuals from and ALC adds individuals to a population, these results seem plausible because the goal is to get rid of rather than to augment pest species.

Since population fluctuations can be particularly important in driving population booms, we have defined outbreaks as the population size exceeding a value well above the carrying capacity, which can only be achieved in deterministic systems if the population cycles. Therefore, our definition of outbreak probability is not simply the inverse of extinction probability, and it differs from related measures such as establishment, invasion or persistence probability, see e.g. [25, 30, 33].

Regarding vulnerable species, our results are similar but reversed. Again, the control intensities of both ALC and ATH need to be high enough to change extinction risk. Once there is an effect, ALC proves beneficial for population persistence and is even able to completely eliminate the collapse risk (large population extinction). By contrast, ATH is either ineffective or counterproductive in preventing outbreaks. These results seem plausible as well because augmenting vulnerable populations appears more suitable than reducing them.

Interestingly, for deterministic population dynamics, we prove that neither ALC nor ATH have any effect on the extinction probabilities of small populations (Proposition 1.1). This makes sense in the case of ATH because it harvests relatively large populations and therefore does not change the production curve at small population sizes. In the case of ALC, its inefficacy may appear surprising at first sight. However, while ALC does restock small populations, it does not do so to large enough a level to exceed the Allee threshold (cf. Fig 2). Hence, the restocking tends to slow down the extinction process, but it cannot prevent extinction in the first place. This could only be achieved by restocking intensities *c* > 1; however, they will cause a population blow-up if implemented also at larger population sizes [11].

In the scenario of large population extinctions, ATH has no effect on the deterministic collapse threshold, whereas ALC is either ineffective as well or can reduce extinction risk depending on the initial condition and the control intensity (Proposition 1.2). This is a surprising result because augmenting the population in this situation is a better option than harvesting it, even though extinction is caused by exceeding a collapse threshold. This can be explained as follows. ATH is ineffective because harvesting takes place only at population sizes below the collapse threshold. ALC can be effective because it restocks populations *after* they have collapsed. Hence, the restocking intervention ‘counter-compensates’ for the overcompensatory population dynamics causing the collapse of large populations. If the order of events or census timing were changed, the quantitative results could be different [40–42].

While the two control methods have no effect in the deterministic small and large population extinction scenarios (or, in the case of ALC, only conditionally), ALC and ATH can become effective (or counterproductive) in the presence of stochasticity. In that sense, stochasticity can be a foe or a friend to management programmes.

The scenario of large population extinction is particularly interesting for another reason. While conservation biology and mathematical modelling has been mostly concerned with small populations [22, 24, 31–33, 43, 44], here we show that also large populations can be at risk of extinction, even if they have population sizes well above the Allee threshold and close to the carrying capacity. At such large population sizes, one might be tempted to expect that Allee effect could be ignored. However, in concert with overcompensatory population dynamics, the population is not safe even at those high levels.

So far, the interplay between Allee effects and overcompensation has been mostly studied in the context of essential extinction [14, 33]. However, as highlighted before, the Allee effect has been largely ignored in control methods aimed at stabilizing populations, but see [11, 45–47]. By contrast, the fisheries literature seems to have paid more attention to the role of Allee effects in managed populations and pointed out that Allee effects curtail yield and stock levels at low population abundance, e.g. [22, 48, 49].

Allee effects have also been found to play a role in biological invasions [50] and in pest outbreaks (e.g., for the gypsy moth see [26, 51]). In this paper, we have studied outbreaks in two different types of models. In the model with a strong Allee effect (Sect. 4), the low-density attractor corresponds to extinction, whereas in the gypsy moth model by Dwyer *et al*. [29] the low-density attractor is positive (Sect. 5). In the latter case, noise promotes even more population variability as it can cause recurrent jumps between attractors.

The results in this paper suggest that ALC can be a suitable control method to enhance persistence stability of small populations or those at risk of extinction, and that ATH can be an effective method for controlling population outbreaks. These conclusions are fundamental for the design of management programmes in biological conservation and pest control. We ought to mention that this paper has not considered other areas of population management. For instance, ATH in particular might be appropriate for the exploitation of biological resources, e.g. in fisheries. To this end, however, both stock stability and yield variability are likely quantities of interest. Regarding ALC, interventions require a stock of individuals that can be added to the controlled population. Hence, ALC might be most appropriate when releasing natural enemies as biocontrol agents, as insect predators or parasitoids may be reared relatively easily. Translocation programmes are another plausible area of application, but the conservation of endangered species may be difficult when the species is rare and cannot be stocked.

## Appendix

In this Appendix we prove the results that have been stated throughout this paper regarding deterministic populations. We denote by

$$\begin{array}{c} R(x)=\max\{f(x),x\cdot c\}\;\text{and}\;H(x)=\min\{f(x),x/h\} \end{array}$$

the production function for populations controlled by ALC and ATH, respectively.

We start by considering *A*_*T*_*c*__ ∈ (*K*, +∞) and *A*_*T*_*h*__ ∈ (0, *K*) as the largest positive solutions of the equations *f*(*x*) = *x* ⋅ *c* for ALC and *f*(*x*) = *x*/*h* for ATH, respectively. These values correspond to the activation thresholds for ALC [7] and ATH [11].

For *x* ∈ (0, *L*) the relative position of the two curves involved in *R*(*x*) may vary with the control intensity, whereas for the other *x*-values *R*(*x*) is completely defined in terms of *A*_*T*_*c*__. For *x* ∈ [*L*, *A*_*T*_*c*__] the curve *y* = *f*(*x*) is above *y* = *x* ⋅ *c* and thus *R*(*x*) = *f*(*x*), whereas for *x* ∈ (*A*_*T*_*c*__, +∞) the relative position of these curves is reversed and then *R*(*x*) = *x* ⋅ *c*.

In the case of ATH, the straight line *y* = *x*/*h* is above *y* = *f*(*x*) for *x* ∈ (0, *L*] and therefore *H*(*x*) = *x*/*h*. For *x* ∈ [*L*, *A*_*T*_*h*__) the relative position of these curves depends on the control intensity, whereas for [*A*_*T*_*h*__, +∞) the curve *y* = *f*(*x*) is above *y* = *x*/*h* and then *H*(*x*) = *f*(*x*).

Graphically, *R* has a bimodal shape with a local maximum at *d* and a local minimum at *A*_*T*_*c*__. *H* is unimodal with a maximum at max{*d*, *A*_*T*_*h*__}.

The next result characterizes the dynamics of the controlled populations when *f*(*f*(*d*)) > *L* holds. This condition is sufficient for bistability under the assumptions (C1)–(C3). It is also necessary in case of a negative Schwarzian derivative, which is true for many population models like the generalized Beverton–Holt, Hassel, quadratic or variants of Ricker [14, 52]. In particular, it is true for the mate-finding Allee effect model considered here for numerical simulations.

**Proposition 1** *Assume that* (*C1*)–(*C3*) *hold and f*(*f*(*d*)) > *L*.

1. *If* $\underset{x\to +\infty }{\text{lim}}f\left(x\right)\geq L$, *uncontrolled populations and populations controlled by ATH and ALC go extinct for x*_0_ ∈ (0, *L*), *and persist for x*_0_ ∈ [*L*, +∞).
2. *If* $\underset{x\to +\infty }{\text{lim}}f\left(x\right)<L$, *there exists a unique U* > *K such that f*(*U*) = *L*. *Uncontrolled populations and populations controlled by ATH persist for x*_0_ ∈ [*L*, *U*] *and go extinct for x*_0_ ∈ (0, *L*) ∪ (*U*, +∞). *Populations controlled by ALC with c* ∈ [*L*/*U*, 1) *persist for x*_0_ ∈ [*L*, +∞) *and go extinct for x*_0_ ∈ (0, *L*), *while for c* ∈ (0, *L*/*U*) *those initiated with* $x_{0}\in \overset{+\infty }{\underset{k=0}{\cup }}\left[L/c^{k},U/c^{k}\right]$ *persist and those with* $x_{0}\in \left(0,L\right)\cup \overset{+\infty }{\underset{k=0}{\cup }}\left(U/c^{k},L/c^{k+1}\right)$ *go extinct*.

*Proof*. We start by proving that small populations starting with *x*_0_ ∈ (0, *L*) go finally extinct in all cases and for all systems. This is obvious for uncontrolled populations, since *f*(*x*) < *x* for all *x* ∈ (0, *L*) and thus orbits correspond to strictly decreasing positive sequences. The limit of these sequences is a fixed point of the system, so must correspond to the extinction state. The same conclusion can be obtained for populations controlled by both ATH and ALC. For *x* ∈ (0, *L*), we have *H*(*x*) = min{*f*(*x*), *x*/*h*} = *f*(*x*) < *x* since *f*(*x*) < *x* < *x*/*h*. On the other hand, *x* ⋅ *c* < *x* and *f*(*x*) < *x* for *x* ∈ (0, *L*), so *R*(*x*) = max{*f*(*x*), *x* ⋅ *c*} < *x*.

Assume now that $\underset{x\to +\infty }{\text{lim}}f\left(x\right)\geq L$. Then, by (C1)–(C3), *f*(*x*) ≥ *L* for all *x* ∈ [*L*, +∞). Consequently, uncontrolled populations that start in [*L*, +∞) persist. This is also true for populations controlled by ALC or ATH. In the case of ALC, the conclusion follows from *R*(*x*) = max{*f*(*x*), *x* ⋅ *c*} ≥ *f*(*x*) ≥ *L* for all *x* ∈ [*L*, +∞). For ATH we must distinguish two cases. For *x* ∈ [*L*, *A*_*T*_*h*__], we have *f*(*x*) ≥ *L* and *x*/*h* ≥ *L*/*h* > *L*, which yields *R*(*x*) = min{*f*(*x*), *x*/*h*} ≥ *L*. On the other hand, *R*(*x*) = *f*(*x*) ≥ *L* for *x* ∈ (*A*_*T*_*h*__, +∞).

Next, assume that $\underset{x\to +\infty }{\text{lim}}f\left(x\right)<L$. The existence of *U* follows by applying Bolzano’s theorem to *h*(*x*) = *f*(*x*) − *L* in (*K*, +∞), and its uniqueness follows by (C3). Moreover, we note that *f*(*f*(*d*)) > *L* = *f*(*U*) yields *f*(*d*) < *U*. We show *f*([*L*, *U*]) ⊂ [*L*, *U*] by considering three different cases. Assume initially *x* ∈ [*L*, *d*]. Then, *L* = *f*(*L*) ≤ *f*(*x*) ≤ *f*(*d*) < *U* because *f* is strictly increasing in (0, *d*). For *x* ∈ (*d*, *f*(*d*)] we have *L* < *f*(*f*(*d*)) ≤ *f*(*x*) < *f*(*d*) < *U*, since *f* is strictly decreasing in (*d*, +∞). The same argument leads to *L* = *f*(*U*) ≤ *f*(*x*) < *f*(*f*(*d*)) < *U* for *x* ∈ (*f*(*d*), *U*] given that *f*(*f*(*d*)) < *f*(*d*) < *U*. This completes all cases and allows us to conclude that uncontrolled populations initiated in [*L*, *U*] persist.

The same conclusion is true for populations controlled by ALC or ATH. For ALC, we have *R*(*x*) = max{*f*(*x*), *x* ⋅ *c*} ≥ *f*(*x*) > *L* for all *x* ∈ [*L*, *U*]. On the other hand, *f*(*x*) ≤ *U* and *x* ⋅ *c* ≤ *U* ⋅ *c* < *U*, yielding *R*(*x*) = max{*f*(*x*), *x* ⋅ *c*} ≤ *U*. For ATH, *H*(*x*) = min{*f*(*x*), *x*/*h*} ≤ *f*(*x*) ≤ *U* for all *x* ∈ [*L*, *U*], and for these values *f*(*x*) ≥ *L* and *x*/*h* ≥ *L*/*h* > *L*, so *H*(*x*) = min{*f*(*x*), *x*/*h*} ≥ *L*.

Populations starting with *x*_0_ ∈ (*U*, +∞) go eventually extinct in the uncontrolled case because *x*_1_ = *f*(*x*_0_) < *f*(*U*) = *L*. Given that *A*_*T*_*h*__ < *K* < *U*, ATH does not alter the production function in the interval (*U*, +∞), and therefore the same conclusion is valid for populations controlled by this method.

Assume now *c* ∈ [*L*/*U*, 1) for ALC. Given that *f*(*U*) = *L* ≤ *U* ⋅ *c*, we have *A*_*T*_*c*__ ≤ *U*. For *x* ∈ (*U*, +∞) we have *R*(*x*) = max{*f*(*x*), *x* ⋅ *c*} = *x* ⋅ *c* > *U* ⋅ *c* ≥ *L*. Since we have already shown that *R*([*L*, *U*]) ⊆ [*L*, *U*], we obtain *R*([*L*, +∞)) ⊆ [*L*, +∞), and hence controlled populations starting in [*L*, +∞) persist.

Finally, consider *c* ∈ (0, *L*/*U*). Since (*L*/*c*) ⋅ *c* = *L* = *f*(*U*) > *U* ⋅ *c* and *f* is strictly decreasing in (*d*, +∞) ⊃ (*U*, +∞), we have *U* < *A*_*T*_*c*__ < *L*/*c*. Controlled populations initiated with *x*_0_ ∈ (*U*, *L*/*c*] go extinct because *f*(*x*_0_) < *f*(*U*) = *L* and *x*_0_ ⋅ *c* < (*L*/*c*) ⋅ *c* < *L*, which yields *x*_1_ = max{*f*(*x*_0_), *x*_0_ ⋅ *c*} < *L*. Consider now *k* ≥ 1 and *x*_0_ ∈ (*U*/*c*^*k*^, *L*/*c*^*k*+1^) ⊂ (*A*_*T*_*c*__, +∞). Then, *x*_*k*_ = *x*_0_ ⋅ *c*^*k*^ ∈ (*U*, *L*/*c*) and thus *x*_*k*+1_ < *L*. This proves that all controlled populations initiated in $\overset{+\infty }{\underset{k=0}{\cup }}\left(U/c^{k},L/c^{k+1}\right)$ go extinct. Assume now *x*_0_ ∈ [*L*/*c*^*k*^, *U*/*c*^*k*^] ⊂ (*A*_*T*_*c*__, +∞) for *k* ≥ 1. Then, *x*_*k*_ = *x*_0_ ⋅ *c*^*k*^ ∈ [*L*, *U*] and hence controlled populations starting in *$\overset{+\infty }{\underset{k=0}{\cup }}\left[L/c^{k},U/c^{k}\right]$* persist.

Next, we show that both ALC and ATH can induce bistable dynamics in an uncontrolled population showing essential extinction.

**Proposition 2** *Assume that* (*C1*)–(*C3*) *hold and the dynamics shows essential extinction*. *Then*, *there exist c*_0_, *h*_0_ ∈ (0, 1) *such that the system controlled by ALC with any intensity c* > *c*_0_ *and the system controlled by ATH with any intensity h* > *h*_0_ *exhibit bistable dynamics*.

*Proof*. According to Proposition 1, it must be *f*(*f*(*d*)) ≤ *L* (otherwise, the dynamics would show bistability). Since *f*(*K*) = *K* > *d* > *L* and *f*(*d*) > *d*, Bolzano’s theorem and the strict decrease of *f* in (*d*, +∞) yield the existence of a unique *U* > *K* verifying *f*(*U*) = *L*. On the other hand, *f*(*x*) ≥ *L* for all *x* ∈ [*L*, *U*] because *f* is strictly increasing in (*L*, *d*), strictly decreasing in (*d*, *U*) and *f*(*L*) = *f*(*U*) = *L*.

Consider the restocking intensity *c*_0_ = *L*/*U* < 1. For *c* > *c*_0_ we can use the same reasoning as in Proposition 1 to show that orbits starting in [*L*, +∞) remain in this interval and the corresponding populations persist.

Let us now study the case of ATH. Consider the harvesting intensity *h* = *d*/*f*(*d*), for which *A*_*T*_*h*__ = *d*. The peak of the stock–recruitment curve for the controlled system is *f*(*A*_*T*_*h*__) = *A*_*T*_*h*__/*h* = *f*(*d*) > *d* and its image is *f*(*f*(*A*_*T*_*h*__)) = *f*(*A*_*T*_*h*__/*h*) = *f*(*f*(*d*)) ≤ *L*. As *h* increases, the straight line *y* = *x*/*h* tends to *y* = *x* and *A*_*T*_*h*__ strictly grows and approaches *K* for *h* → 1. Then, given that *f* is strictly decreasing in (*d*, +∞), the term *f*(*A*_*T*_*h*__) = *A*_*T*_*h*__/*h* strictly decreases and tends to *f*(*K*) = *K*. With the same argument, *f*(*A*_*T*_*h*__/*h*) strictly increases and tends to *K* > *L*. Hence, according to Bolzano’s theorem, there must exist *h*_0_ ≥ *d*/*f*(*d*) such that *f*(*A*_*T*_*h*_0___/*h*_0_) = *L*. Moreover, given the strict increase of *f*(*A*_*T*_*h*__/*h*), we have *f*(*A*_*T*_*h*__/*h*) > *L* for *h* > *h*_0_. By arguments already used here, we obtain *H*(*L*, *U*) ⊂ [*L*, *U*], and we conclude that the system controlled by ATH shows bistability for *h* > *h*_0_.
